# Coagulase-Negative Staphylococci Contained in Gut Microbiota as a Primary Source of Sepsis in Low- and Very Low Birth Weight Neonates

**DOI:** 10.3390/jcm9082517

**Published:** 2020-08-04

**Authors:** Edyta Golińska, Magdalena Strus, Anna Tomusiak-Plebanek, Grażyna Więcek, Łucja Kozień, Ryszard Lauterbach, Dorota Pawlik, Beata Rzepecka-Węglarz, Jolanta Kędzierska, Małgorzata Dorycka, Piotr B. Heczko

**Affiliations:** 1 Jagiellonian University Medical College, 31-121 Cracow, Poland; edyta.golinska@uj.edu.pl (E.G.); a.tomusiak@uj.edu.pl (A.T.-P.); grazyna.wiecek@uj.edu.pl (G.W.); lucja.kozien@uj.edu.pl (Ł.K.); piotr.heczko@uj.edu.pl (P.B.H.); 2 Department of Neonatology, Jagiellonian University, Medical College, 31-501 Cracow, Poland; ryszard.lauterbach@uj.edu.pl (R.L.); dorota.pawlik@uj.edu.pl (D.P.); 3 Department of Neonatal Intensive Care, “UJASTEK” Medical Centre, 31-752 Cracow, Poland; b.rzepecka@wp.pl; 4 Department of Microbiology, University Hospital, 31-501 Cracow, Poland; jkedzierska@su.krakow.pl; 5 Microbiological Laboratory, Diagnostics Inc. Krakow Branch, 31-864 Cracow, Poland; malgorzata.dorycka@diag.pl

**Keywords:** sepsis, CoNS, staphylococcus, newborn, neonates, translocation

## Abstract

Background: There are only a few reports in the literature about translocation of coagulase-negative staphylococci (CoNS) as a primary cause of sepsis in neonates, although CoNS are among a short list of “translocating” bacteria when present in abundance. Methods: 468 blood samples, 119 stool samples, and 8 catheter tips, from 311 neonates, were tested for presence of microorganisms. CoNS strains isolated from the blood and stool or from blood and catheter tip of the same newborn at approximately the same time were paired and typed with PFGE (Pulse-Field Gel Electrophoresis) method. The strains were then tested for the presence of adherence genes and biofilm formation. Results: The strains with identical PFGE profiles in comparison to those with non-identical profiles differed in terms of the pattern of the virulence genes and showed a lack of the genes related to adherence, but more often presence of IS256, which is related to virulence. They also were phenotypically unable to adhere to intestinal Caco2 cells. Conclusions: A considerable proportion of CoNS strains isolated from bloodstream of VLBW/LWB neonates was identical to the strains isolated from faeces of the same neonates at the same time. These observations may offer indirect evidence indicating that at least some CoNS can translocate from the gastrointestinal tract of the premature neonates into the bloodstream and thus cause generalized infection.

## 1. Introduction

Sepsis in low-birth-weight (LBW) and very low-birth-weight (VLBW) neonates is still one of the most significant causes of neonatal morbidity and mortality. Owing to the immature immune system and exposure to many invasive diagnostic and therapeutic procedures, LBW/VLBW neonates are at the highest risk for bacterial sepsis, with a prevalence of 1 to 5 per 1000 live births in developed countries [1,2]. The spectrum of bacterial pathogens of neonatal sepsis varies with the geographical area and time. Currently, in developed countries, coagulase-negative staphylococci (CoNS), such as *S. epidermidis* or *S. haemolyticus*, are the major pathogens involved in late-onset sepsis [3,4,5]. Data from the Polish Neonatology Surveillance Network also confirm this tendency. In the years 2009–2011, among microorganisms isolated from 1695 infants with VLBW, Gram-positive cocci, and mostly CoNS, predominated over other microorganisms [6].

It is commonly accepted that CoNS are able to induce clinically significant bacteraemia because of their natural niche on human skin and their ability to adhere to biomaterials and to form biofilm. Biofilm formation is the key mechanism of CoNS pathogenesis, particularly in relation to catheter-related infections [7]. It is believed, although not substantially documented, that single cells of the large CoNS populations present on the skin surface adhere to catheter material, colonize it, migrate along the catheter down through the wound and adjacent tissues to blood vessel lumen, and then multiply in the bloodstream [8]. Consistent with some reports, another possible pathomechanism of sepsis in LBW and VLBW neonates is translocation of bacteria from gut lumen to bloodstream due to increased intestinal permeability typical for premature neonates [9,10,11]. Some studies have demonstrated that Gram-negative bacteria can be translocated through the gut wall and reach the bloodstream either directly by invading submucosal capillary vessels or indirectly via Peyer’s patches and lymphatics and cause bacteraemia and then sepsis [12,13,14].

There are only a few reports in the literature about translocation of CoNS as the primary cause of sepsis in neonates [15], although staphylococci and especially CoNS, according to some authors, are part of the short list of “translocating” bacteria when present in abundance [16,17].

Therefore, the aim of this study was to find evidence supporting the hypothesis that the primary source of the CoNS causing sepsis in LBW and VLBW neonates treated in neonatal intensive care units (NICU) is not only their skin but also gut microbiota. For this purpose, we used a standardized molecular typing PFGE method to compare DNA profiles of CoNS strains isolated from paired blood and stool samples of LBW and VLBW neonates with clinical signs of sepsis. Moreover, for selected CoNS strains we performed the adherence assay and tested for the presence of genes coding for different virulence factors.

## 2. Materials and Methods

### 2.1. Patients

A total of 311 LBW and VLBW neonates hospitalized in the Department of Neonatology, University Hospital, Jagiellonian University Medical College in Cracow and in the Department of Neonatal Intensive Care, Ujastek Medical Center in Cracow, in the years 2016–2017 with microbiologically confirmed sepsis, were included in the study. Neonates were monitored in the cooperating centers and appropriately cared for according to standards of medical care for neonates provided by the Polish Society of Neonatology. Late-onset sepsis (LOS) was defined according to criteria published by Gastmeier [18]:

- Presence of at least two of the following: temperature > 38 °C or <36.5 °C or temperature instability, tachycardia or bradycardia, apnea, prolonged capillary refill, metabolic acidosis, hypoglycaemia, other signs of bloodstream infections such as lethargy;
- Recognized pathogen cultured from one or more blood cultures or CoNS isolated from at least one blood culture or intravascular line and one of the following: C-reactive protein > 2.0 mg/dL, immature/total neutrophil ratio (I/T ratio) > 0.2, leukocytes < 5000/μL, platelets < 10,000/μL.

### 2.2. Ethics Approval

This study was approved by the Bioethics Committee of Jagiellonian University Medical College no. 122.6120.222.2015.

### 2.3. Consent Form


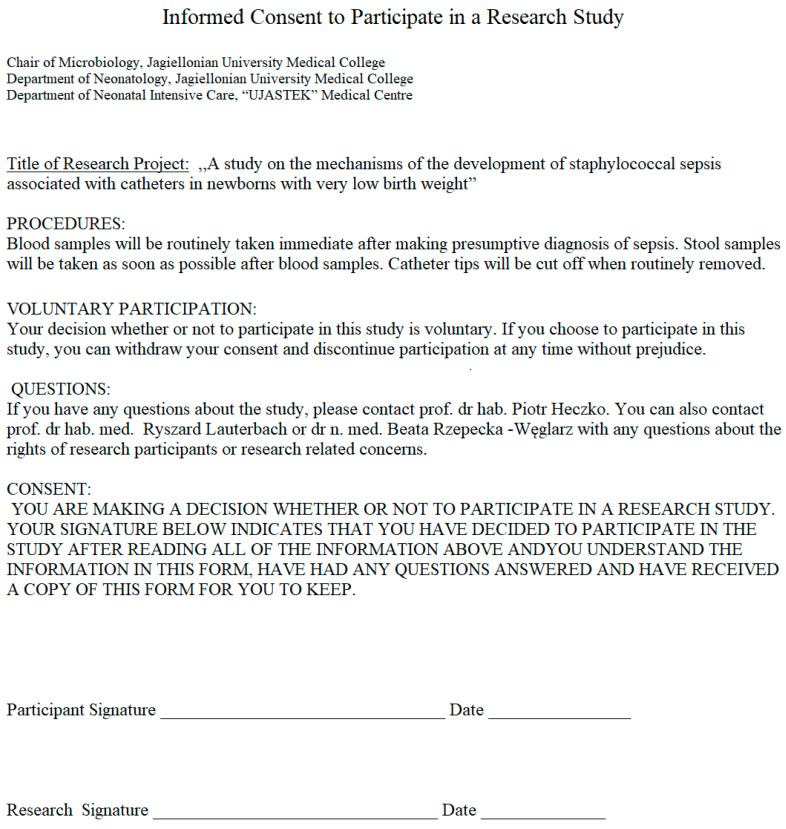


## 3. Microbiological Cultures

### 3.1. Blood Samples

Blood samples were routinely taken immediately after making presumptive diagnosis of sepsis and transported to the university hospital microbiological laboratory (UHML) or microbiological laboratory of the Diagnostyka Ltd., Cracow, Poland. Aseptically collected blood specimens of at least 1 millilitre volume were injected into an aerobic blood culture bottle (Bactec Plus 26 Aerobic; BD Microbiology Systems), incubated, and then subcultured on MacConkey agar, horse blood agar (at 37 °C, each for 24 h), and Sabouraud agar (at 37 °C for 38 h). The isolates were identified using mass spectrometry (MALDI Biotyper, Bruker Scientific LLC, Billerica, MA, USA) according to manufacturer’s instructions. Actual MBT IVD Library of mass spectra was applied. All isolates identified into species belonging to CoNS were transported to the Chair of Microbiology, Jagiellonian University Medical College (CM) for further investigations. They were checked for purity and kept frozen for further studies.

### 3.2. Stool Samples

Stool samples were collected from neonates with suspected sepsis as soon as possible after collecting blood samples. They were placed in transport media provided by the CM and kept frozen at −70 °C until transported to CM. The frozen samples were then homogenized in 1 mL of Schaedler’s broth (SAB). The samples were plated on the following media: McConkey Agar (Oxoid, Hampshire, UK) for Enterobacteriaceae, Columbia Blood Agar (Difco, CA, USA) with 5% sheep blood for staphylococci and streptococci and Enterococcosel Agar (BBL, MD, USA) for enterococci. The plates were incubated at 37 °C aerobically for 24 h. The morphology of the grown colonies was analyzed under magnifying glass, and several colony picks of each morphological type were subcultured on appropriate aerobic and anaerobic media and Gram-stained. Phenotypic identification was performed using commercial identification systems (API 20E, API20A, APIStaph, APIStrept (bioMerieux, l’Etoile, France).

All CoNS isolates were then speciated by mass spectrometry (as above) and kept frozen for further studies.

### 3.3. Catheter Tips

Catheter tips routinely removed from vessels close to the time of suspected sepsis were cut off by sterile scissors, placed in sterile tubes, and kept frozen at −70 °C until transported to CM. The catheters were checked for the presence of adherent bacteria using a semiquantitative roll-plate technique described previously by Maki, by transferring each catheter tip onto a plate with Columbia agar supplemented with 5% sheep blood and rolling the tip back and forth across the surface at least 3 to 4 times [19]. Colonies grown on roll-plates were identified as described above. All isolates initially identified as CoNS were speciated by mass spectrometry (as above), collected, and kept frozen for further studies.

## 4. Molecular Typing

Pulse-Field Gel Electrophoresis (PFGE) was used as the standard molecular typing technique for CoNS [20]. A protocol developed for CoNS and used in our laboratory on previous occasions was employed [21]. Differences in banding patterns were documented by Gel Compar (AppliedMaths, Sint-Martens-Latem, Belgium) using the Dice coefficient and unweighted pair group method with arithmetic mean. Isolates with more than 95% similarity were clustered together as identical.

## 5. PCR Amplification of Genes Coding for Virulence Factors

PCR (Polymerase Chain Reaction) technique was used to show presence or absence of the 5 genes coding virulence factors (*icaA*, *sesI*, *sesD*/*bph*, *Hld*, and IS256) attributed to biofilm formation, adherence to human epithelium and mucus, and host cell damage. The PCR reactions were performed in a BioRad (Hercules, CA, USA) thermocycler. Amplification was performed according to methodologies previously described [22,23,24,25,26]. Primers and PCR conditions are listed in Table 1.

## 6. Adherence of CoNS to CaCo2 Cells

The ability of the CoNS strains to adhere to the human gut epithelium was checked in vitro. Human intestinal epithelial cell line CaCo2 from colon carcinoma (American Type Culture Collection, Middlesex, United Kingdom) was used. The cells were grown in Dulbecco modified Eagle medium (DMEM)/F-12 (GIBCO, Carlsbad, CA, USA) supplemented with 10% heat-inactivated foetal bovine serum (FBS) (GIBCO). The cells grew to confluence on 0.4-m semipermeable tissue culture inserts (Transwell; Corning, NY, USA) in a humidified incubator at 37 °C and 5% CO_2_. Briefly, 48h culture of Caco-2 cells at a density of 1 × 10^6^ cells/mL were cultivated for 24 h in a 12-well flat bottom tissue culture plate (Iwaki, Japan) on Eagle’s 1959 medium (MEM; Biomed, Lublin, Poland) with L-glutamine and NaHCO_3_ (IITD, Wrocław, Poland) containing 5% fetal calf serum (Sigma-Aldrich Chemie, Germany) and antibiotics (penicillin 100 UI/mL, streptomycin 100 UI/mL, neomycin 200 μg/mL) (Sigma Aldrich Chemie, Germany) and then washed twice with PBS. Overnight cultures of bacteria were diluted with MRS+MEM to a concentration of ~10^8^ CFU/mL. Cells in wells were inoculated with bacterial culture. After incubation at 37 °C for 30 min, wells were washed twice with PBS to release unbound bacteria. Then, the cells were fixed with 3.7% formaldehyde for 1h, washed twice with PBS, and stained with crystal violet stain (Merck, Germany). The adherent bacterial cells were counted in 20 randomly selected microscopic fields. Adhesion degree was evaluated using a semiquantitative score system, from (0) to (3), as used by us before [27] and based on the following legend:

(a) strong adherence (3): >80 bacterial cells per field
(b) moderate adherence (2): 61–80 bacterial cells per field
(c) weak adherence (1): 41–60 bacterial cells per field
(d) no adherence (0): <40 bacterial cells per field

All experiments were run in duplicate.

## 7. Phenotypic Characteristic of Slime Production Ability on Congo Red Agar (CRA)

All the strains were cultured on CRA plates. Plates were prepared by adding 0.8 g of Congo red (Sigma, St. Louis, MO, USA) and 36 g of saccharose (Sigma, St. Louis, MO, USA) to 1 L of brain heart infusion agar (Oxoid, Basingstoke, Hampshire, UK). Tested strains were incubated for 24 h at 37 °C on CRA plates. Slime-producing strains form black and very black colonies, whereas non-producing strains develop red and bordeaux colonies, according to scale proposed by Arciola [28].

## 8. Statistical Analyses

Statistical analyses were performed to demonstrate significant differences in the presence of selected genes, and in the degrees of adherence and biofilm formation among tested CoNS strains. The Fisher exact test was used.

## 9. Results

Altogether 468 blood samples, 119 stool samples and 8 catheter tips from 311 neonates, were tested for presence of microorganisms. CoNS were the most commonly isolated group of bacteria, followed by Gram-negative rods (Table 2). In total, 443 CoNS were isolated. Of these, 368 originated from blood, 69 from stool, and 6 from catheter tips. Among CoNS strains, these belonging to *S. epidermidis* species predominated over *S. haemolyticus*, *S. capitis*, and *S. hominis*; only a few strains belonged to other species.

Other bacteria isolated from blood samples were represented by 111 Gram-negative rods cultivated from all samples: 79 from blood and 32 from stool samples. They belonged to *E. coli* (22.5%), *K. pneumoniae* (22.5%), and *K. oxytoca* (20%) species. Except for Gram-negative rods, various *Streptococcus* spp. and *Candida* spp. strains were found.

**Figure 1:**
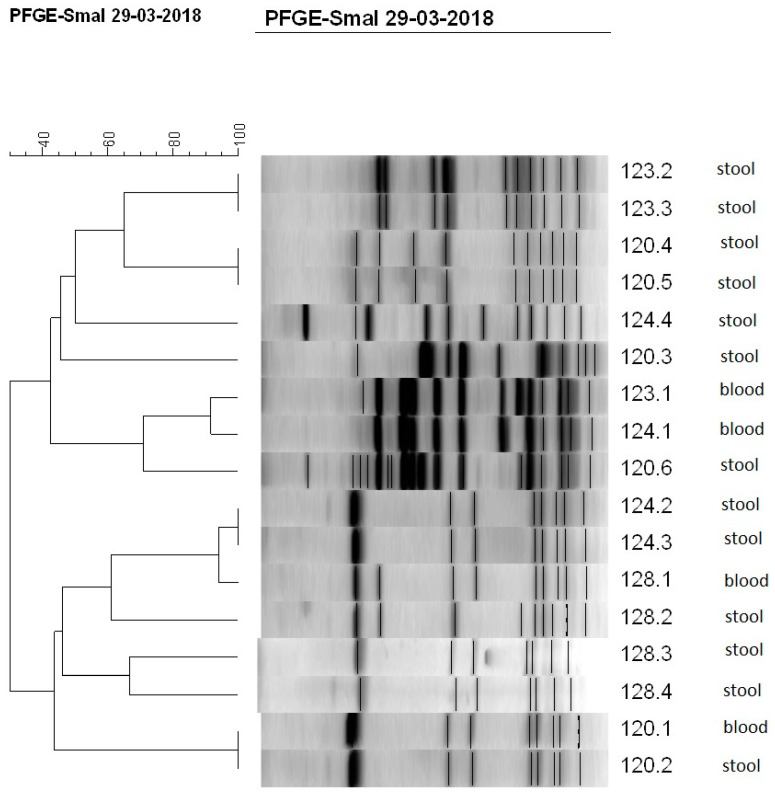
Dendrogram showing the percent similarity between CoNS isolated from blood and stool of four newborns (120, 123, 124, and 128) at the same time. Isolates were digested with SmaI restriction enzyme. PFGE band profiles are shown, and the scale indicates percent similarity.

Further, CoNS strains isolated from the blood and stool or from blood and catheter tip of the same newborn at approximately the same time were paired and typed with PFGE method. Identity of the strains was confirmed using criteria of Tenover et al. [29]. Altogether, 69 pairs of CoNS strains isolated from blood and stool samples, and 6 CNS pairs isolated from blood and catheter tips, were compared (Table 3, Figure 1). It appeared that 26% of CoNS pairs from blood-stool group showed identical profile (pulsotype), which indicates that the same strain was isolated from blood as from stool. The rest of the pairs had different profiles, which means that different strains were isolated from blood and stool. No identical profiles were found for pairs from blood and catheter tips. The most common CoNS species occurring in pairs with the same pulsotype was *S. haemolyticus* (67%), followed by *S. epidermidis* and *S. capitis*, while in pairs with non-identical profile it was *S. epidermidis* (55% in blood-stool group, and 67% in blood-catheter tips group). Other pairs with non-identical profile belonged to *S. warneri*, *S. hominis* species, and CoNS strains not readily identified.

**Figure 2:**
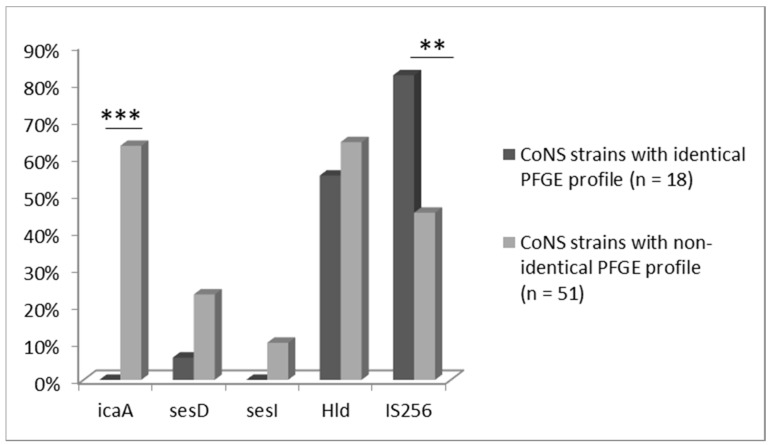
Proportional occurrence of tested virulence genes in CoNS strains (*n* = 69) isolated from blood, which have identical (*n* = 18) and non-identical (*n* = 51) PFGE profile with strains isolated from the stool samples (*** *p* < 0.001; ** *p* < 0.01).

Occurrence of genes coding for adherence and biofilm formation in pairs of CoNS strains of the same or different PFGE profiles isolated from blood and stool samples was then analyzed (Figure 2). While identifying genes coding for selected virulence factors (*icaA*, *sesD*, *sesI*, *Hld*, IS256) in the genomes of CoNS strains, statistically significant differences in the frequencies of the *icaA* (*p* < 0.001) and IS256 (*p* < 0.01) in pairs of identical versus non-identical strains were confirmed. *icaA* gene, responsible for the synthesis of adhesin forming the biofilm matrix, and *sesI*, encoding proteins responsible for colonization and adherence, were detected only in strains from blood-stool pairs with a non-identical PFGE profile. Moreover, *sesD* gene, also responsible for adherence, was found in 11/51 strains with non-identical profile, whereas it was present in 1/18 strains in strains with identical profile. Hld gen was commonly found in most tested stains. On the other hand, sequence IS256 attributed to virulence was more frequently found in identical pairs of the strains than in non-identical ones.

**Figure 3:**
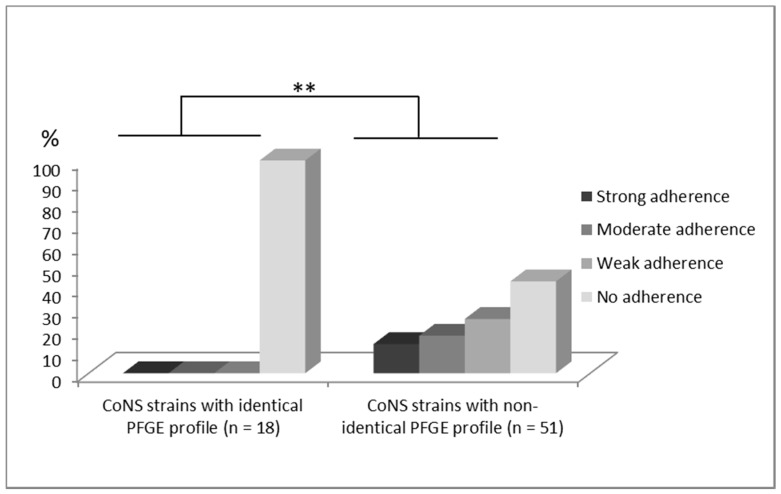
Comparison of degrees of adherence to Caco 2 cells evaluated in a semi-quantitative scale (0–no adherence, 1–weak adherence, 2–moderate adherence, 3–strong adherence) (** *p* < 0.01) between two tested CoNS groups isolated from blood, which have identical (*n* = 18) and non-identical (*n* = 51) PFGE profile with strains isolated from stool samples.

Adherence assay was performed on all 69 tested strains. It was observed that the strains from blood and stool with identical PFGE profiles showed a complete lack of adherence to CaCo2 tissue (100%). On the contrary, strains with non-identical profiles presented ability to adhere in high proportions: 13.7% of strains showed strong, 17.6% intermediate, 25.5% weak adherence, and 43.1% lack of adherence. Statistically significant difference in adherence between both groups was confirmed (*p* = 0.0014) (Figure 3).

The analyzed strains were examined for their ability to form biofilm. There were no biofilm-forming strains in CoNS group strains with identical PFGE profile. Only 9 of 51 strains from non-identical PFGE profiles group were able to produce biofilm. There were no statistically significant differences between tested groups.

## 10. Discussion

It is commonly accepted that bacteraemia and subsequent sepsis related to catheters inserted into blood vessels to deliver drugs in children treated in neonatal intensive care units (NICU) is caused by bacteria, mainly CoNS, which are members of the skin microbiota [30,31]. This hypothesis was partially confirmed in our study, where CoNS were the most commonly identified bacteria in the blood of premature newborns with low and very low birth weight and confirmed sepsis. However, the results of the PFGE typing, which enabled comparison of genetic profiles of the strains isolated from bloodstream and GI tract of the same neonates, showed that a small proportion of CoNS from these two sources had identical profiles. This may suggest that some of the CoNS translocate from the gastrointestinal tract into the bloodstream and thus cause generalized infection. The ability of CoNS to penetrate the intestinal barrier and translocate is not fully supported by literature data, but some researchers suggest that CoNS may have this attribute [17,32]. At the same time, no strain among those isolated from catheter tips showed identical profile with strains from the bloodstream. This may indicate that CoNS-colonizing catheters, which are probably derived from the skin microbiota, are not etiological CoNS strains related to sepsis.

It should be stressed here that in spite of the fact that complexity of the gut microbiota in premature neonates is not very high [33], isolation of CoNS strains from faeces to make pairs with their bloodstream-derived counterparts is difficult, and thus it may be so that a proportion of such strains has not been found in faecal cultures and in practice sepsis cases caused by gut-derived CoNS are more numerous.

Most common species of CoNS strains isolated from blood samples belonged to *S. epidermidis* and *S. haemolyticus*; however, the majority of the paired and identical strains were represented by *S. haemolyticus*, which may suggest that this species more often than other CoNS possess some mechanisms enabling them to translocate and cause a generalized infection. Our results are in accordance with studies of Soeorg et al. [17], in which 10 of 11 studied *S. haemolyticus* showed the genotypic similarity between bloodstream and gut isolates. The same group [34] reported later that *S. haemolyticus*, causing sepsis in neonates, originated mainly from intestines. They also demonstrated that mother’s breast milk is the main source of neonatal gut colonization with CoNS.

Another evidence supporting the hypothesis that neonatal staphylococcal sepsis may be caused by CoNS strains colonizing gut of preterm infants comes from molecular studies on their genes related to their ability to adhere and form biofilm versus those attributed to virulence.

*IcaA* gene is responsible for the ability to create biofilm and adhere to host cells and artificial surfaces. The presence of this gene is an important factor in the virulence of CoNS. *SesD* gene, also called *bhp*, codes for a surface protein that facilitates bacterial adhesion to host tissue. Thus, these genes are responsible for facilitation of the colonization and spread in the hospital environment [25]. *SesI* gene is responsible for the intensity of virulence, but also affects initial adhesion and encodes proteins from the sortase group. Microorganisms carrying this gene show increased resistance to antibiotics and host immune factors, and are more easily present in the hospital environment [35]. A group of proteins encoded by the *Hld* gene cause damage to the host cell membrane, leading to necrosis and apoptosis of the polynucleated and mononuclear cells. Thus, presence of this gene facilitates the proliferation and colonization abilities of the pathogenic CoNS strains [36,37]. IS256 is a sequential fragment of the entire gene, responsible for virulence. Its action is based on the reversible transposition of genes responsible for the production of biofilm or regulating its production [38]. It is believed that IS256 is associated with virulence determinant genes, contributes to genetic adaptation, and is active in invasive strains during infection. Expression of *ica* operon may be affected by IS256. IS256 increases the production of PIA (polysaccharide intercellular adhesin), which plays an important role in biofilm formation and immune system invasion [39,40]. Previous studies have reported IS256 as an important factor for *S. epidermidis* infectivity and a marker to differentiate the invasive and commensal *S. epidermidis* isolates [41,42,43].

In this study, CoNS strains isolated from bloodstream and identical with their counterpart strains isolated in parallel from faeces possessed no *icaA* gene. Presence of a *sesD* gene also responsible for adherence was shown only in 5.5% of strains belonging to this group. On the other hand, these strains, unlike others with discrepant PFGE profiles, i.e., unrelated to gut colonization, showed, in vast majority of cases, the presence of IS256.

The lack of the genes, responsible for adhesion to host cells and tissues, seems to allow CoNS to enter the bloodstream and cause infection. The results of our molecular studies on presence of the adherence genes in CoNS were also confirmed by our phenotyping studies on adherence to CaCo 2 cells. Strong and intermediate adherence to human cell line were more common for strains with non-identical PFGE profile, whereas strains with compatible profile mostly showed lack of adherence. We obtained similar results when examining group A streptococci from invasive infections: the most virulent strains showed no or very poor adherence to human cell cultures [26]. Maybe, in the future, checking for the absence of *icaA* in addition to identification and testing the drug resistance profile of CoNS isolated from newborns will be used as markers indicating high probability of sepsis.

It has been demonstrated that *S. epidermidis* sessile cells growing in biofilm in vivo and in vitro show different gene expression than the same but planktonic cells, and that this process is regulated by Fe-dependent mechanism [44]. It is thus probable that CoNS present in biofilm on gut mucosa may be liberated during its decomposition [45] after Fe-signalling, and then they translocate across a weak gut-blood barrier in premature neonates. Alternatively, specific CoNS cells present in gut and armoured with genes enabling tissue penetration but not biofilm formation may directly pass the barrier and cause sepsis. Such a possibility was shown in the study of Soeorg et al. [34] on *S. haemolyticus* mecA-positive and IS256-positive virulent strains originating from mother’s milk and causing sepsis in premature neonates. Our studies also seem to support this hypothesis.

Studies on the mechanisms of CoNS utilized to evoke sepsis in premature newborns with low and very low birth weight may offer practical approaches in neonatal sepsis prevention. A broader assessment of the virulence markers of CoNS with a consistent genetic profile in blood and faeces may allow for a more accurate understanding of how these strains enter the bloodstream, and thus prevent sepsis by effective control of their colonization and translocation. For example, in our previous clinical study on the effects of administration of the probiotic *L. rhamnosus* and *B. breve* we found that gut colonization of preterm neonates with the latter bacteria resulted in a significant decrease of the CoNS-related sepsis rates. This observation offered indirect evidence that gut colonization with CoNS poses a risk of the staphylococcal neonatal sepsis in VLBR and LBR infants that may be decreased by extensive neonatal gut colonization with bifidobacteria [46].

## 11. Conclusions

One-quarter of CoNS strains isolated from bloodstream of VLBW/LWB neonates were identical to the strains isolated from faeces of the same neonates at the same time as demonstrated by PFGE profiling. The majority of the strains with identical PFGE profiles in this study belonged to *S. haemolyticus* genus.

The strains with identical PFGE profiles in comparison to those with non-identical profiles differed in pattern of the virulence genes and especially showed a lack of the genes related to adherence; indeed, they were phenotypically unable to adhere to intestinal Caco2 cells.

On the other hand, the strains with identical PFGE profiles more often showed the presence of IS256, which is related to virulence, than those with non-identical profiles.

These observations may offer indirect evidence indicating that at least some CoNS can translocate from the gastrointestinal tract of the premature neonates into the bloodstream and thus cause generalized infection.

Transparency declaration.

## Figures and Tables

**Table 1:** Primer sequences and polymerase chain reaction (PCR) conditions for various genetic markers.

Gene	Forward Primer (5′→3′)	Reverse Primer (5′→3′)	Product Size (bp)	PCR Conditions	References
**icaA**	AAA CTT GGT GCG GTT ACA GG	TCT GGG CCT GAC CAT GTT G	750	1 min 94 °C 30 s 57 °C 1 min 72 °C 39 cycles	[26]
**IS256**	TGAAAAGCGAAGAGATTCAAAGC	ATGTAGGTCCATAAGAACGGC	1102	1 min 94 °C 1 min 59 °C 90 s 72 °C 34 cycles	[23]
**sesD**	TGCCAATCATCAAACCACTG	GTCACTGAACCGATTAACCCACTT	635	1 min 94 °C 1 min 50 °C 1 min 72 °C 30 cycles	[25]
**sesI**	GCT GAT TAT GTA AAT GAC TCA AAT	AGC TTT TGT TGT TTG AGC TTC	389	1 min 95 °C 1 min 49 °C 1 min 72 °C 34 cycles	[22]
**Hld**	ATG GCA GCA GAT ATC ATT TC	CGT GAG CTT GGG AGA GAC	444	2 min 94 °C 1 min 45 °C 1 min 72 °C 29 cycles	[24]

**Table 2:** Microbial species isolated from blood, stool, and catheter tips from neonates with sepsis.

Etiological Factors:	Number of Isolates (*n* = 511) from 468 Blood Samples	Number of Isolates (*n* = 148) from 119 Stool Samples	Number of Isolates (*n* = 7) from 8 Catheter Tips
Coagulase-negative staphylococci	**368 (72%)**	**82 (55.4%)**	**6 (85.7%)**
*S. epidermidis*	164	47	4
*S. haemolyticus*	80	16	1
*S. capitis*	58	2	0
*S. hominis*	30	0	0
*S. lugdunensis*	2	0	0
*S. warneri*	2	1	0
*S. xylosus*	1	0	0
*S. caprae*	1	0	0
CNS	30	16	1
Coagulase-positive staphylococci	**15 (2.9%)**	**4 (2.7%)**	**1 (14.3%)**
*S. aureus*	15	4	1
Gram negative rods	**79 (15.5%)**	**32 (21.6%)**	**0**
*Klebsiella* spp.	41	4	0
*E. coli*	16	9	0
*S. marcescens*	9	0	0
*E. cloace*	7	0	0
*P. mirabilis*	3	0	0
*A. baumannii*	1	0	0
*S. linguefaciens*	1	0	0
*P. aeruginosa*	1	0	0
*other*	0	19	0
Other (*Micrococcus* spp., *Streptococcus* spp., *Candida* spp.)	**49 (9.6%)**	**30 (20.3%)**	**0**

**Table 3:** Identity of the paired coagulase-negative staphylococci (CoNS) strains isolated from blood, catheter tips, and stool of the same patient at the same time, as compared with the Pulse-Field Gel Electrophoresis (PFGE) method.

	Sample Pairs (Blood + Stool) Taken from the Same Newborn (*n* = 69)			Sample Pairs (Blood + Catheter Tips) Taken from the Same Newborn (*n* = 6)		
		PFGE			PFGE	
	Total Number	Identical Profile	Not Identical Profile	Total Number	Identical Profile	Not Identical Profile
Coagulaso negative staphylococci	**69**	**18**	**51**	**6**	**0**	**6**
*S. epidermidis*	32	4	28	4	0	4
*S. haemolyticus*	22	**12**	10	1	0	1
*S. capitis*	10	2	8	0	0	0
*S. hominis*	2	0	2	0	0	0
*S. warneri*	1	0	1	0	0	0
*CNS*	2	0	2	1	0	1
